# New Insights in the Hydrothermal Synthesis of Rare-Earth Carbonates

**DOI:** 10.3390/ma12132062

**Published:** 2019-06-27

**Authors:** Luca Spiridigliozzi, Claudio Ferone, Raffaele Cioffi, Mauro Bortolotti, Gianfranco Dell’Agli

**Affiliations:** 1 Department of Civil and Mechanical Engineering, University of Cassino and Southern Lazio, Via G. Di Biasio 43, 03043 Cassino (FR), Italy; 2 Department of Engineering, Università di Napoli “Parthenope”, Centro Direzionale, Isola C4, 80143 Napoli, Italy; 3 INSTM—National Interuniversity Consortium of Materials Science and Technology, Via G. Giusti 9, 50121 Florence, Italy; 4 Department of Industrial Engineering, University of Trento, via Sommarive, 9, 38123 Trento, Italy

**Keywords:** rare-earth carbonates, hydrothermal treatment, powder morphology, tengerite-type structure

## Abstract

The rare-earth carbonates represent a class of materials with great research interest owing to their intrinsic properties and because they can be used as template materials for the formation of other rare earth phases, particularly of rare-earth oxides. However, most of the literature is focused on the synthesis and characterization of hydroxycarbonates. Conversely, in the present study we have synthesized both rare-earth carbonates—with the chemical formula RE_2_(CO_3_)_3_·2-3H_2_O, in which RE represents a generic rare-earth element, and a tengerite-type structure with a peculiar morphology—and rare-earth hydroxycarbonates with the chemical formula RECO_3_OH, by hydrothermal treatment at low temperature (120 °C), using metal nitrates and ammonium carbonates as raw materials, and without using any additive or template. We found that the nature of the rare-earth used plays a crucial role in relation to the formed phases, as predicted by the contraction law of lanthanides. In particular, the hydrothermal synthesis of rare-earth carbonates with a tengerite-type structure was obtained for the lanthanides from neodymium to erbium. A possible explanation of the different behaviors of lighter and heavier rare-earths is given.

## 1. Introduction

Rare-earth compounds have drawn much research attention in the last few years, emerging as good functional materials with a wide range of applications [1]. Very recently, a book edited by Martin-Ramos and Ramos Silva described the state-of-the-art of lanthanide-based multifunctional materials, focusing on the importance of lanthanides for novel key-enabling technologies [2]. Among rare-earth compounds, those that are rare-earth carbonate-based, including carbonates, hydroxycarbonates, and oxycarbonates, represent a very important class. On one hand they are potential materials for specialized industrial applications such as the lighting industry, catalysis, and as a matrix for luminescence materials etc. [3,4], and on the other hand, they can be used as a template or as sacrificial materials for the synthesis of other nanocrystalline rare earth phases, especially oxides [1] but also oxysulfides [5]. A further field of research concerning lanthanides carbonates focuses on the complex formation among Ln^3+^ and carbonate ions and is aiming to develop a model for chemical migration of actinide ions in the natural aquifers near a nuclear waste repository [6]. Thus, several studies in the last few years have reported findings concerning the rare-earth carbonates; for example, Vallina et al. [3,7] studied Dy, La, and Nd carbonate crystallization from amorphous products, and Ganguly et al. [4] synthesized micro flower structures of europium hydroxycarbonate by homogeneous precipitation. Furthermore, amorphous doped and codoped cerium hydroxycarbonates with nanosized spherical morphology, which proved to be excellent precursors of ceramic electrolytes in Intermediate Temperature Solid Oxide Fuel Cells (IT-SOFC), were synthesized by controlled coprecipitation using ammonium carbonate as the precipitating agent [8,9,10,11]. Pourmortazavi et al. [12] synthesized uniform spherical nanopowders of sacrificial neodymium carbonate by precipitation. Li et al. [13] synthesized LaCO_3_OH, with various morphologies using various precipitation processes, suitable as a precursor of doped La_2_O_2_CO_3_/La_2_O_3_ nano/microcrystals, with interesting optical properties. In addition, as further confirmation of the great interest in rare-earth carbonates, first Kaczmarek et al. [1] and then Kim et al. [14] very recently authored two systematic reviews concerning the synthesis, properties, and applications of this class of materials. 

Hydrothermal treatment is a well-known method for synthesizing nanosized functional ceramic materials with tunable morphology; some of its most relevant applications are in the synthesis of zirconia-based ceramics [15,16,17], ceria-based ceramics [18,19], perovskite-type structure RECrO_3_ [20], and titania-based ceramics [21] etc. In several previous studies [18,22,23], we showed that hydrothermal treatment at 120 °C of products obtained by mixing rare-earth nitrate solution with ammonium carbonate solution caused the formation of carbonate-based crystalline phases with substantial differences in terms of composition, crystal structure, and powder morphology, as a function of the nature of the rare-earth used, the molar ratio between carbonate anions and rare-earth cations (this parameter is indicated by “R” in the following), and of the duration of the hydrothermal treatment. Keeping in mind the great interest in cerium-based products, in these works we mainly focused on cerium carbonates, both doped and codoped, by systematically investigating the effects of time and R-value. Therefore, continuing these studies, in the present work we aimed to analyze the hydrothermal synthesis of other rare-earth carbonates in detail, focusing especially on the carbonates in order to detect both their stability, as a function of the rare-earth used, and their best synthesis conditions. In fact, whilst there are a lot of reports concerning the synthesis of rare-earth hydroxycarbonates (see for example [3,4,7,8,9,10,11,12,14,18,22,23]), to the best of our knowledge there are very limited studies focused on the synthesis methods and characterization of rare-earth carbonates, especially those with a tengerite-type structure. Furthermore, most of these studies are rather outdated [24] and often report contradictory results, and discussions, concerning the compositions and properties of the rare-earth carbonates, caused by a lack of structural data [25]. As further confirmation, even in the overview of rare-earth carbonate crystal structures found in the Inorganic Crystal Structure Database (ICSD) database and reported by Kaczmarek et al. [1], the only card related to a tengerite-type carbonate is the one for Y_2_(CO_3_)_3_·2H_2_O.

Our results show that, as a function of the rare-earth used and the duration of hydrothermal treatment, it is possible to synthesize rare-earth hydroxycarbonates, hydrated rare-earth carbonates, biphasic products and even amorphous phases. In particular, with the proposed hydrothermal treatment it is possible to synthesize (in the absence of any additive) the hydrated rare-earth carbonate, RE_2_(CO_3_)_3_·2-3H_2_O with a tengerite-type structure with a very peculiar morphology, for all the rare-earths, ranging from neodymium to erbium and also for yttrium, through careful selection of the duration of the treatment. The effects of the nature of the rare-earth and the duration of hydrothermal treatment were systematically studied and a possible mechanism of the transformations involving the rare-earth carbonates is proposed.

## 2. Materials and Methods

Rare-earth (III) nitrates (i.e., RE(NO_3_)_3_·xH_2_O 99.9% from Sigma Aldrich, Milan, Italy, with x = 5 or 6 depending on the rare-earth), as metal precursors, and ammonium carbonate ((NH_4_)_2_CO_3_ 99.0%, Fluka-Honeywell, Sigma-Aldrich, Steinheim, Germany), as the precipitating/mineralizing agent, were used as raw materials for the hydrothermal syntheses. All the chemicals were used as received without any further purification.

Regardless of the rare-earth precursor used, the procedure for the hydrothermal syntheses was always the same, and it is described as follows:

(a) The proper amount of rare-earth nitrate was dissolved in deionized water to obtain a 0.1 M solution (solution A) and ammonium carbonate was dissolved in deionized water to obtain a 0.5 M (solution B). Both solutions were vigorously stirred for 1 h to favor the homogenization.
(b) The proper volume of solution B was quickly added to the selected volume of solution A, maintained under mild stirring, in order to reach R = 2.5, where R is the molar ratio between carbonate ions and rare-earth cations. When solution B was added to solution A, a white precipitate formed instantly.
(c) The as-prepared suspensions were immediately transferred into Teflon vessels (60 mL), which were then sealed and held in outer stainless-steel pressure vessels for the hydrothermal treatment. The treatment was carried out in an air-thermostatted rotating oven at 120 °C and 25 rpm to allow the complete homogenization of the system during the process.
(d) After the selected reaction times, the vessels were quenched with cold water and the resulting products were recovered by vacuum filtration, repeatedly washed with deionized water, and finally dried overnight at 80 °C in static air. The various synthesized samples were identified with a label reporting the rare-earth symbol and the duration of the treatment; as an example, sample Pr8h indicates the sample was obtained using Praseodymium nitrate as the precursor, with R = 2.5, and hydrothermally treated at 120 °C for 8 h.

Several durations of the hydrothermal treatment were selected as a function of the rare-earth used, varying in the range 2–210 h. Furthermore, some selected experiments were carried out with the same procedure but using R = 10 as molar ratio carbonate/rare-earth cation, with the aim of highlighting the effect of a large excess of carbonate anions. In this case R10 was added to the label of the sample, so the label Yb210hR10 indicates the sample was obtained using Ytterbium nitrate as the precursor, with R = 10, and hydrothermally treated at 120 °C for 210 h.

All samples were characterized by X-ray powder diffraction (XRD) using a diffractometer Miniflex 600 (Rigaku, Tokyo, Japan) to detect the crystalline phases. The lattice parameters for the various samples were extracted by the unit cell refinement procedure using the ReX 0.9.0 Rietveld refinement software [26]. Instrumental broadening was characterized by means of an Y_2_O_3_ standard (99.9% from Sigma Aldrich, Milan, Italy) assuming a Pseudo-Voigt peak profile convoluted with an axial-divergence asymmetry function [27]. Average volume-weighted crystallite size was calculated by means of the standard integral breadth model described in [28], in the approximations of pure Lorentzian size broadening and isotropic crystallite shape. Tengerite-type phases were modeled starting from the reference Y_2_(CO_3_)_3_·2H_2_O crystal structure reported in [29]; peak intensities were directly extracted in the refinement by using an empty cell, symmetry-constrained model as described in [30]. Hydroxycarbonate phases, on the other hand, were simulated starting from the REOHCO_3_ crystal structures in the literature [31,32] and replacing the RE atom in the structure according to the compound of interest.

The thermal behavior of the samples was investigated through simultaneous differential thermal analysis (DTA) and thermogravimetric analysis (TG) with a TGA/DSC 2 STAR^e^ analyzer (Mettler-Toledo, Columbus, OH, USA) in air, with a heating rate of 10 °C/min up to 1200 °C and α-Al_2_O_3_ was used as a reference.

The morphology of the powders was observed with a Scanning Electron Microscope (SEM) Phenom ProX equipped with an Energy Dispersive X-ray Spectrometry (EDS) system (Phenom-World BV, Eindhoven, The Nederland).

## 3. Results

**Figure 1:**
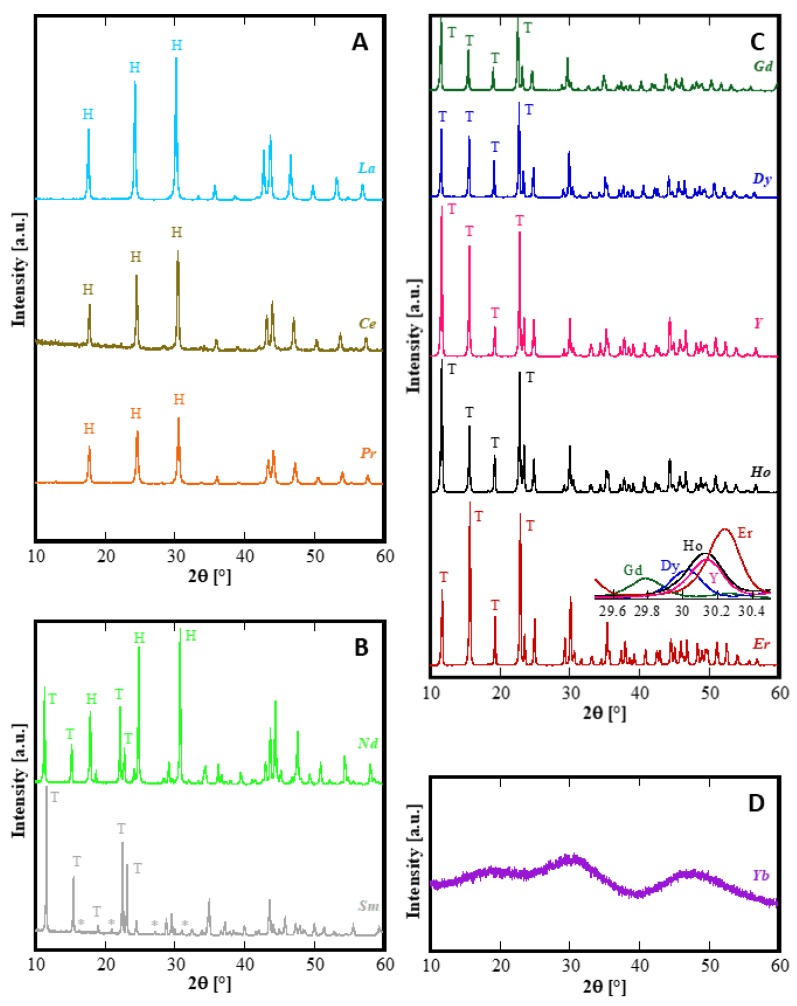
Diffraction patterns of samples synthesized at 8 h; the patterns are grouped into four panels in which the behavior is analogous. T, stands for the phase with a tengerite-type structure and, H, stands for hexagonal-hydroxycarbonate. (**A**): La, Ce and Pr (only H-phase); (**B**): Nd and Sm (two-phases products); (**C**): Gd, Dy, Y, Ho and Er (only T-phase); (**D**): Yb, amorphous phase. The inset in Figure 1C is the direct comparison related to the (123) peak of tengerite-type crystal structure.

As also reported in the literature [10,33], regardless of the actual rare-earth nitrate used in the precipitation, the as-formed precipitates, i.e., the precursors of the hydrothermal syntheses, were all amorphous or nearly amorphous in nature and formed by nanosized spherical-like particles bundled in rounded clusters. During the hydrothermal treatment they went through very different crystallization paths and morphological modifications as a function of both RE^3+^ cation and the aging duration. Figure 1 shows the diffraction patterns of all the samples treated for 8 h. For sake of clarity, Figure 1 is divided into four panels each reporting one typical behavior exhibited after 8 h of treatment: type-A behavior characterized by the presence of one crystalline phase (“H”) related to La, Ce, Pr (Figure 1A); type-B behavior with the presence of two crystalline phases, “H” and “T” for Nd and “H” and “*” for Sm (Figure 1B); type-C behavior characterized by the presence of one crystalline phase (“T”) related to Gd, Dy, Ho, Er and Y (Figure 1C); and finally, type-D behavior related to Yb (Figure 1D). 

**Figure 2:**
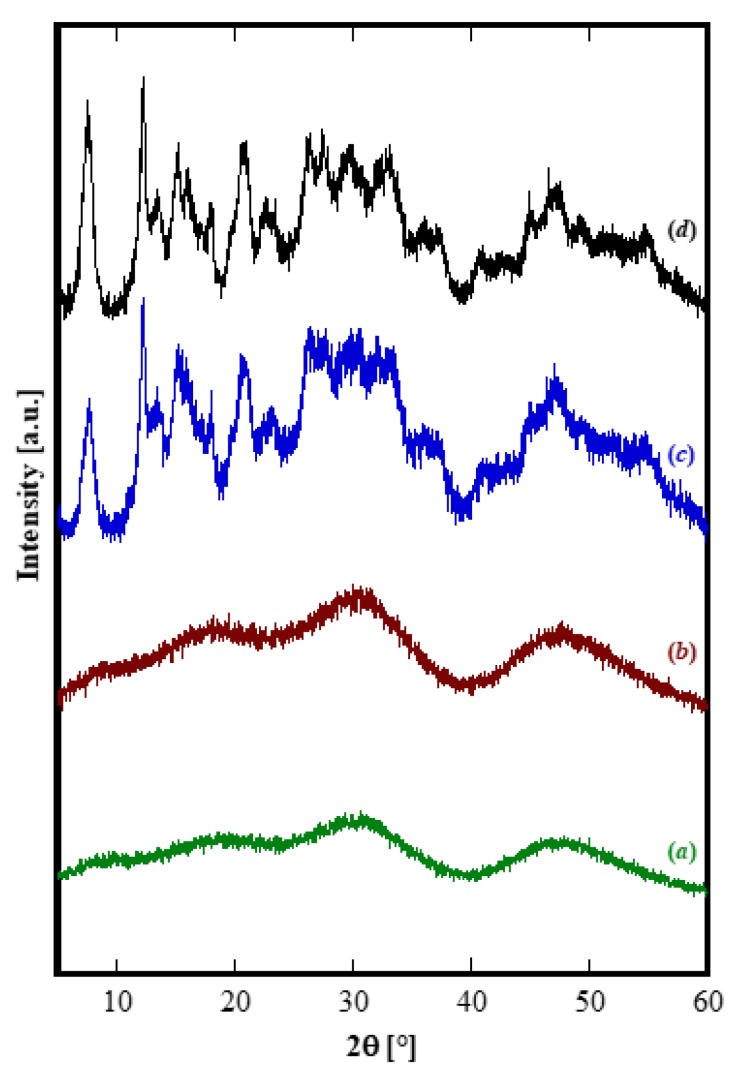
Diffraction patterns of Yb-based products for several durations: (**a**) 8 h with R = 2.5, (**b**) 48 h with R = 2.5, (**c**) 48 h with R = 10, and (**d**) 210 h with R = 10.

**Figure 3:**
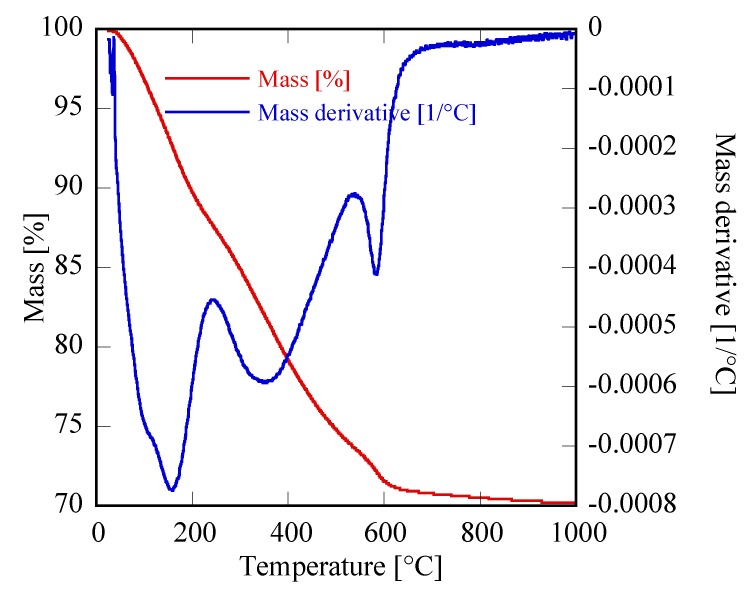
Thermogravimetric (TG) and TG derivative of sample Yb48hR10.

First of all, it can be observed that, with the exclusion of Yb, for all other RE the amorphous precursor fully crystallizes in a short time, i.e., within 8 h, during the hydrothermal treatment at 120 °C. On one hand, these results seem interesting because in the literature the hydrothermal crystallization of rare-earth carbonates is generally reported at higher temperatures and often at longer times than those used in this work [34,35]. On the other hand, the different behavior found for ytterbium compared to the other rare-earths is frequently reported in literature [14,36]. Thus, the Yb-based precursor did not show any crystallization after an aging of 8 h (Figure 1D). In order to induce the hydrothermal crystallization of some Yb carbonate-based compound, we extended the treatment duration up to 48 h, in the same conditions, however, the sample was still amorphous (See Figure 2b). Furthermore, by using a large excess of ammonium carbonate, i.e., with R = 10, after 48 h only incipient crystallization occurred and a nearly identical result was obtained even prolonging the treatment to 210 h, (see Figure 2c,d, respectively). Due to the very broad peaks and the large overlap with the amorphous halos present in the pattern, the phase identification is very difficult. However, on the basis of the TG and of the DTG, reported in Figure 3, it could be inferred that sample Yb48R10 is a hydrated yttrium carbonate, with the formula Yb_2_(CO_3_)_3_·2H_2_O; in fact, the weight loss measured in Figure 3 is 29.7%, a value very close to the theoretical one equal to 29.8%, and the thermal decomposition occurred in three events, as typical for hydrated rare-earth carbonate, even if no hypothesis on the crystal structure of that phase could be formulated. 

All the rare-earths belonging to the group with type-C behavior (Gd, Dy, Ho, Er and Y) exhibit a very similar diffraction pattern (see Figure 1C), the only differences being a non-negligible shift in the peaks position, better highlighted in the inset at about 30° 2θ, and some dissimilarities in the relative intensity of the XRD peaks. As is well known, Y does not belong to the lanthanides, but is included in the rare-earth group because of its similar properties. As confirmation of this, the hydrothermal behavior of the Y-based precursor was very similar to that of the Gd, Dy, Ho and Er precursors, and the fact that the ionic radius of Y^3+^ (0.1019 nm in VIII coordination) is included between Ho^3+^ (0.1015 nm in VIII coordination) and Dy^3+^ (0.1027 nm in VIII coordination) is further strong evidence of the central role of the ionic radius on the hydrothermal transformations. Focusing on the Y-pattern in Figure 1C, we see that all present peaks can be assigned to the hydrated yttrium carbonate, with the chemical formula Y_2_(CO_3_)_3_·2H_2_O, ICDD, International Centre for Diffraction Data, card number 81-1538, a mineral known as tengerite-(Y) [30]. Although, to the best of our knowledge, corresponding cards do not exist in the current database of crystal structures for the other rare-earth carbonates with a tengerite-type structure, it can be reasonably assumed that for Gd, Dy, Ho, and Er, hydrated normal carbonates analogous to Y have also been formed via the hydrothermal process. Actually, in a rather old work by Wakita and Nagashima [24], crystallographic data for several rare-earth carbonates attributed to a tengerite-type structure were reported; however they do not seem reliable, based on those of tengerite-(Y) (compare data reported in [24], with data in [30] and in the ICDD card number 81-1538). However, a detailed analysis of the structural features of the samples in Panel C was outside the aim of this work and will be addressed in a forthcoming study. For the purpose of the present study, the precise determination of the lattice parameters of the tengerite-type structure was sufficient in order to link the ionic radii with the lattice parameters for the rare-earths showing C-behavior. As the term tengerite-(RE) has been approved only for RE = Y, in the following for indicating the hydrated RE_2_(CO_3_)_3_ with a tengerite-type crystal structure we use the notation “tengerite-(RE)” (note the double quotations) for RE other than Y. The crystallographic data of the samples in Figure 1, Panel C, determined using the software ReX are reported in Table 1. The unit cell edges are slightly different from the ones of tengerite-(Y), yet perfectly justifiable considering the difference in ionic radius among Y^3+^ and the other rare-earth cations. 

**Figure 4:**
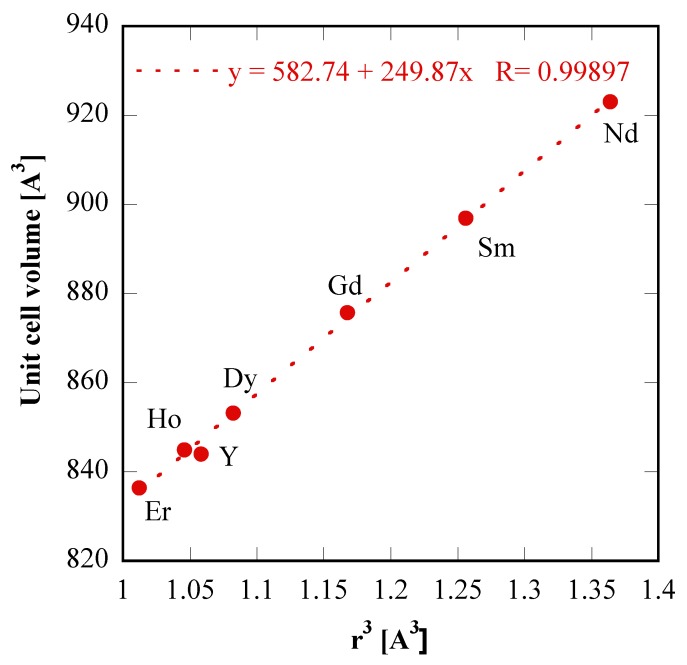
Unit cell volumes of “tengerite-(RE)” rare-earth carbonates as a function of ionic radius raised to cubic power.

In order to assess the global consistence of the data in Table 1, in which the lattice parameters of “tengerite-(Nd)” and of “tengerite-(Sm)” are also reported (see below for discussion about treatments on Nd and Sm precursors), the plot of unit cell volume as a function of the rare-earth ionic radius raised to cubic power is displayed in Figure 4; the resulting nearly perfect linear regression confirms further the validity of the hypothesis that all these materials share the same crystal structure, i.e., a tengerite-type structure.

**Figure 5:**
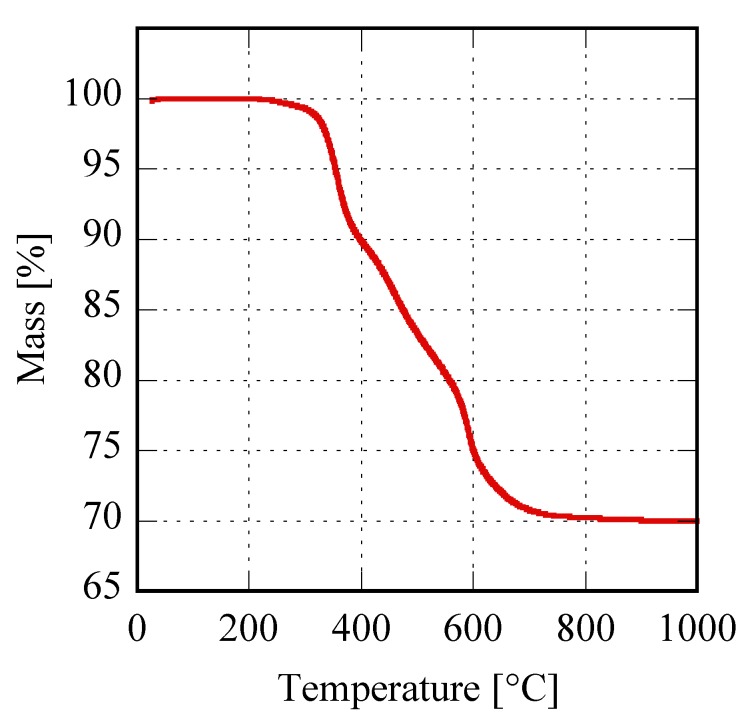
Thermogravimetric plot of sample Er8h.

Finally, the thermal behavior of these materials was highlighted via TG analysis, with the corresponding thermogravimetric plots very similar for all the samples. In Figure 5 a representative sample is shown, the plot related to Er8h which exhibits a global weight loss of 30.1%, divided into three distinct steps perfectly compatible with the following thermal decomposition mechanism proposed for the rare-earth carbonates [14]:

$${\mathrm{Er}}_{2}{\left({\mathrm{CO}}_{3}\right)}_{3}\cdot 2H_{2}O\to {\;\mathrm{Er}}_{2}{\left({\mathrm{CO}}_{3}\right)}_{3}+2H_{2}O↑$$

$${\mathrm{Er}}_{2}{\left({\mathrm{CO}}_{3}\right)}_{3}\to {\;\mathrm{Er}}_{2}O_{2}{\mathrm{CO}}_{3}+2{\mathrm{CO}}_{2}↑$$

$${\mathrm{Er}}_{2}O_{2}{\mathrm{CO}}_{3}\to {\;\mathrm{Er}}_{2}O_{3}+{\mathrm{CO}}_{2}↑$$

**Figure 6:**
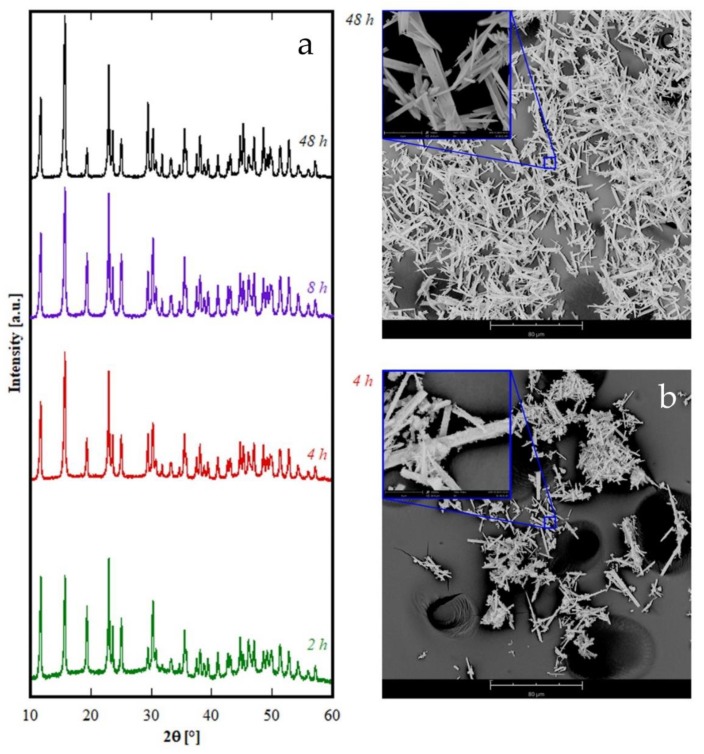
Diffraction patterns (**a**) and SEM micrographs of samples containing Er synthesized at 4 h (**b**) and 48 h (**c**).

The theoretical total weight loss of the previous reactions, i.e., 30.5%, was in very good agreement with the measured value; furthermore, the partial weight losses associated with the three distinct decomposition steps, clearly visible in Figure 5, agree well with the values corresponding to the reported mechanism, for which the theoretical weight losses are in the order of 6.5%, 16.0% and 8.0%. The other samples in Figure 1C show very similar TG plots (not reported here) exhibiting weight loss very close to the theoretical one, even if the water molecules can be slightly different from 2 (for example in the case of sample Gd8h, the “tengerite-(Gd)” has 2.5 water molecules). This point is not surprising as on one hand the water content in a product obtained starting from an aqueous solution can slightly vary as a function of several parameters [37], and on the other hand, the chemical formula of rare-earth carbonates is generally reported as RE_2_(CO_3_)_3_·2-3H_2_O [30]. Therefore, we can suppose that the rare-earths with ionic radius included in the range 0.1–0.106 nm form via hydrothermal treatment, in our chemical-physical conditions, a hydrated carbonate with the chemical formula RE_2_(CO_3_)_3_·2-3H_2_O and a tengerite crystal structure. No other phase appeared through decreasing or increasing the duration of the treatment, except that when the duration was too brief the amorphous precursor was not able to fully crystallize, as happened, for example, for Er with a 2 h treatment. To display the behavior of rare-earths in Figure 1C as a function of the duration of the hydrothermal treatment, the diffraction patterns of the Er-based precursor at various times are displayed in Figure 6. After 2 h, the sample is crystallized in the tengerite-type structure, even though the presence of some residual amorphous phase clearly appears; to highlight the presence of the amorphous halos in the diffraction patterns, the intensity axis in Figure 6 is in square root scale. Also, after 4 h a small amount of amorphous phase is probably present, whereas for longer durations “tengerite-(Er)” is fully crystallized. These results allow us to suppose that the phase with the tengerite-type structure is the equilibrium one—or maybe a metastable phase but with very slow transformation kinetics— in the chemical–physical conditions of the hydrothermal treatment. Figure 6 also shows the morphology of the samples obtained with Er after 4 h and 48 h, these micrographs perfectly confirming the diffraction data. In fact, “tengerite-(Er)” is constituted by very large acicular particles (see micrograph at 48 h in Figure 6), with a length of several tenths of micrometers, width of some micrometers, and thickness of only a few hundreds of nanometers (see also inset in SEM micrograph of Er48h). This peculiar morphology remains essentially unchanged from 2 h up to 48 h, although with increasing treatment duration a noticeable increase of crystal size is found from the diffraction patterns. In fact, average volume-weighted crystallite size values, as refined in the Rietveld analysis, are 266, 320, 484 and >1000 nm for 2 h, 4 h, 8 h and 48 h samples respectively, indicating remarkable grain growth occurring during the hydrothermal aging. It needs to be pointed out that these values are indicative of the average dimension of perfectly coherent (defect-free) crystalline domains, and are not necessarily correlated with the macroscopic particle size as observed by SEM. In fact, the acicular particles are very likely polycrystalline aggregates experiencing, however, internal crystallite growth or coalescence with treatment time. However, when a residual amorphous phase is present, as in samples Er2h and Er4h, this phase exhibits a very different morphology. In fact, by careful analysis of the SEM micrograph of sample Er4h, two different types of particles can be observed; i.e., the acicular particles associated with “tengerite-(Er)”, and the extremely small and rounded particles covering the acicular particles, associated with the amorphous phase (see also inset in SEM micrograph of Er4h). This hypothesis is supported by the absence of the small rounded particles in samples Er48h as well as by evidence in the literature [10,11]. These results suggest that the very large particles of “tengerite-(Er)” probably grew via a step-by-step mechanism in which the amorphous particles of precursor were dissolved, exploiting their relatively higher solubility, and then reprecipitated on the surface of the acicular particles, thereby enlarging them.

**Figure 7:**
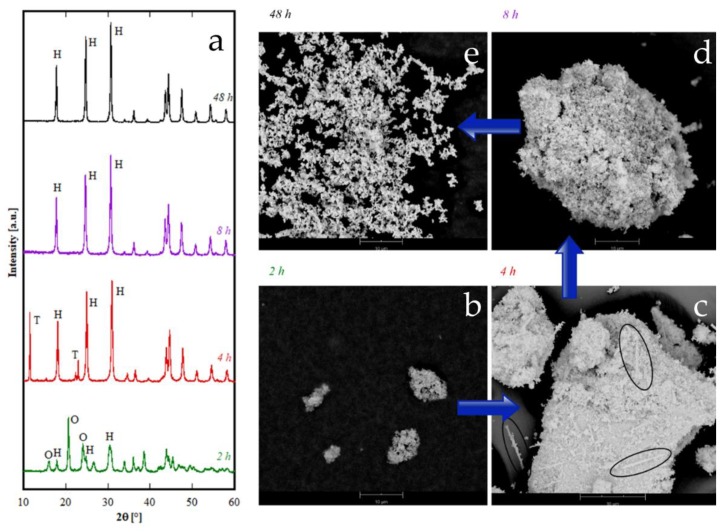
Diffraction patterns (**a**) and SEM micrographs of samples containing Pr synthesized at various durations: 2 h (**b**), 4 h (**c**), 8 h (**d**) and 48 h (**e**). T stands for “tengerite-(Pr)” phase, H stands for hexagonal-hydroxycarbonate and O stands for orthorhombic-hydroxycarbonate.

The rare-earths with the largest ionic radius, i.e., La, Ce, and Pr whose cationic radius is larger than 0.1126 nm, are all crystallized as hexagonal hydroxycarbonate h-RECO_3_OH after 8 h, as evident in the corresponding diffraction patterns in Figure 1A. In fact, all the XRD peaks found for La, Ce, and Pr can be assigned to ICDD card numbers 62-0030, 62-0031, and 62-0024 respectively, which are all related to the corresponding hexagonal hydroxycarbonate, with space group number 174 [31,32,38]. As further confirmation, the lattice parameters determined by unit cell refinement are shown in Table 2, and they are all very close to the theoretical values reported in the corresponding ICDD cards; in addition, the cell volume decreases with the decreasing cationic radius. When varying the duration of the treatment, some rather complex behavior emerged, especially for short duration hydrothermal treatment; in the case of Ce this has been described in great detail in [23] whereas in the case of Pr, it is disclosed by the diffraction patterns in Figure 7. Samples Pr2h and Pr4h (see corresponding diffraction patterns in Figure 7) are very probably both biphasic, with one phase being h-PrCO_3_OH in both the samples. The second phase for sample Pr4h can be reasonably associated with “tengerite-(Pr)” according to the main peak at about 11° 2θ and the two peaks at about 22° 2θ, all marked with a “T” in Figure 7 and also with the presence of acicular particles in the morphology of this sample (see corresponding SEM micrograph in Figure 7b–e). In contrast, the presence in the diffraction pattern of Pr2h of all the main peaks of orthorhombic PrCO_3_OH, ICDD card number 26-1349, clearly suggest that the second phase in sample Pr2h is such a polymorph. However, when prolonging the treatment beyond 8 h, the only phase present for Pr is h-PrCO_3_OH. The morphology of Pr-based samples is revealed by SEM micrographs displayed in Figure 7 in correspondence with the diffraction patterns. Very interesting considerations can be drawn from them. Firstly, the equilibrium phase, i.e., h-PrCO_3_OH, is formed by very small spherical-like particles bundled in rounded clusters which tend to break with as the hydrothermal treatment proceeds (compare SEM for Pr48h and Pr8h), confirming previous results related to h-CeCO_3_OH prepared with the same process. Furthermore, for short durations the hydrothermally synthesized products are biphasic, and consequently, in addition to the rounded particles, in the morphology of sample Pr4h some acicular-like particles also appear (see SEM for Pr4h in Figure 7 in which some acicular particles are highlighted by ovals), reasonably attributable to the minority “tengerite-(Pr)” phase. However, in sample Pr2h, no evident morphological differences among the particles appear, indicating that, due to the rather incomplete crystallization of the sample (see Figure 7) related to the short duration of the treatment, o-PrCO_3_OH and h-PrCO_3_OH show a similar morphology, very probably resembling that of the precursor. In summary, the phase transformations occurring in Pr-based precursors seem to involve two metastable phases, an orthorhombic hydroxycarbonate [32] (indicated by “O” in Figure 7b) and the “tengerite-(Pr)” (indicated by “T” in Figure 7c), that quickly transform into hexagonal hydroxycarbonate which is the only phase present after 8 h.

**Figure 8:**
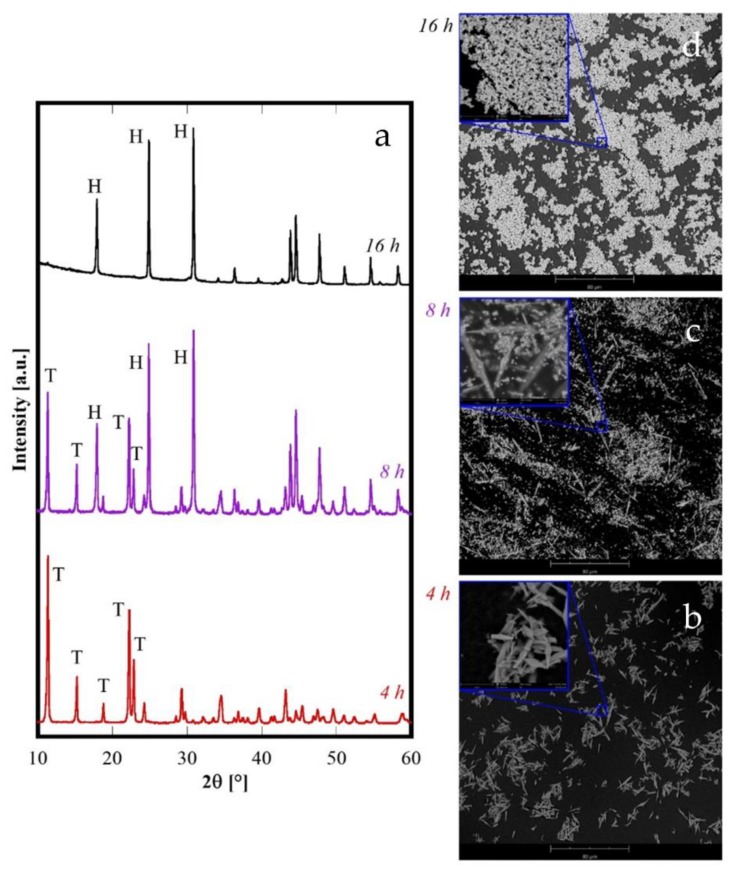
Diffraction patterns (**a**) and SEM micrographs of samples containing Nd synthesized at various durations: 4 h (**b**), 8 h (**c**) and 16 h (**d**). T stands for tengerite phase and H stands for hexagonal-hydroxycarbonate.

Finally, a further different situation is obtained for Nd and Sm (Figure 1B). Both Nd8h and Sm8h are formed by two crystalline phases: the carbonate with a tengerite-type structure, and the hydroxycarbonate. For Nd8h the presence of two phase is evident, but also for Sm8h careful inspection of its diffraction pattern reveals the presence of a second phase in addition to the “tengerite-(Sm)”. In fact, the small XRD peaks marked by the “*” in Figure 1B, patterns related to Sm, are attributable to orthorhombic SmCO_3_OH, isostructural to orthorhombic NdCO_3_OH (ICDD card number 27-1296). Therefore, the type-B behavior is intermediate to type-A and type-C. The behavior of Nd was analyzed in more detail and the results related to 4 h and 16 h, under the same conditions of hydrothermal treatment, are displayed in Figure 8. Here, the diffraction pattern of Nd4h clearly reveals that it is constituted only by “tengerite-(Nd)” whereas the one for Nd16h shows only the peaks of h-NdCO_3_OH. Thus, the phase transformations during the hydrothermal aging of Nd-precursor at 120 °C are perfectly congruent with its intermediate position in the rare-earth series. Nd behavior can be summarized as follows: the first formed phase is the hydrated carbonate, then it evolves rather quickly into hydroxycarbonate and after 16 h this last transformation is completed. The morphological evolution, highlighted by the SEM micrographs displayed in Figure 8b–d, entirely confirms this hypothesis. In fact, the sample Nd4h is formed only by acicular particles with a length of some tenths of micrometers (see 4 h micrograph in Figure 8b) whereas the sample Nd16h is formed only by submicrometric spherical-like particles (see 16 h micrograph in Figure 8d); in contrast, the sample Nd8h is formed by both acicular particles and spherical-like ones.

## 4. Discussion

The crystallization path during the hydrothermal treatments carried out in the present study (T = 120 °C, R = 2.5, dilute solutions etc.) strongly depends on the selected rare-earth. However, by carefully analysing the results, and considering the behavior of rare-earth carbonates in aqueous solution, a comprehensive interpretation of the whole set of results can be proposed. As is well-known [10], at ambient conditions lighter rare-earths (La-Eu) form octahydrate carbonates with a lanthanite structure which readily hydrolyse to hydroxycarbonate, while the heavier rare-earths carbonates have tengerite-type structures which are more resistant to hydrolysis. Conversely, at temperatures close to 100 °C, all rare-earth carbonates should hydrolyse in water, being hydroxycarbonates in the stable phases. Furthermore, on one hand the carbonates have K_sp_ much lower than those of the corresponding hydroxycarbonates [10], and on the other hand the morphology modifications occurring between the amorphous precursors and the corresponding crystalline products clearly suggest a dissolution-precipitation mechanism for the hydrothermal crystallization. Thus, taking all the above into account, we can suppose that irrespective of the actual rare-earth present in the amorphous precursor, the first step of the hydrothermal crystallization is the formation of non-equilibrium phases, such as orthorhombic hydroxycarbonates or hydrate carbonate, as suggested by the present results (see Figure 1, Figure 7 and Figure 8) and also considering the results reported in [23].

In some cases (La, Ce, Pr) these non-equilibrium phases are very quickly converted, i.e. within 8 h of hydrothermal aging, into the corresponding hydroxycarbonates. In other cases (from Gd to Er), this transformation is extremely slow, or even does not occur at all, whilst the acicular particles grow at the expense of the amorphous phase via a step-by-step mechanism; as a consequence, we were not able to observe the formation of the hydroxycarbonates. In intermediate cases (Nd and Sm) the hydrolysis rate of carbonate is slow enough that after aging for 8 h more phases could be detected. In addition, the diffraction pattern of Sm after 8 h (Figure 1B) suggests that its behavior is more similar to the samples with C-behavior, although further confirmation is necessary, whereas the behavior of Nd closely resembles that of Pr, only with a lower transformation rate (compare Figure 8c with Figure 7c). Finally, in the case of Yb, the amorphous phase is also stable for very long durations of treatment, possibly because of the very slow dissolution kinetics of Yb-based compounds.

**Figure 9:**
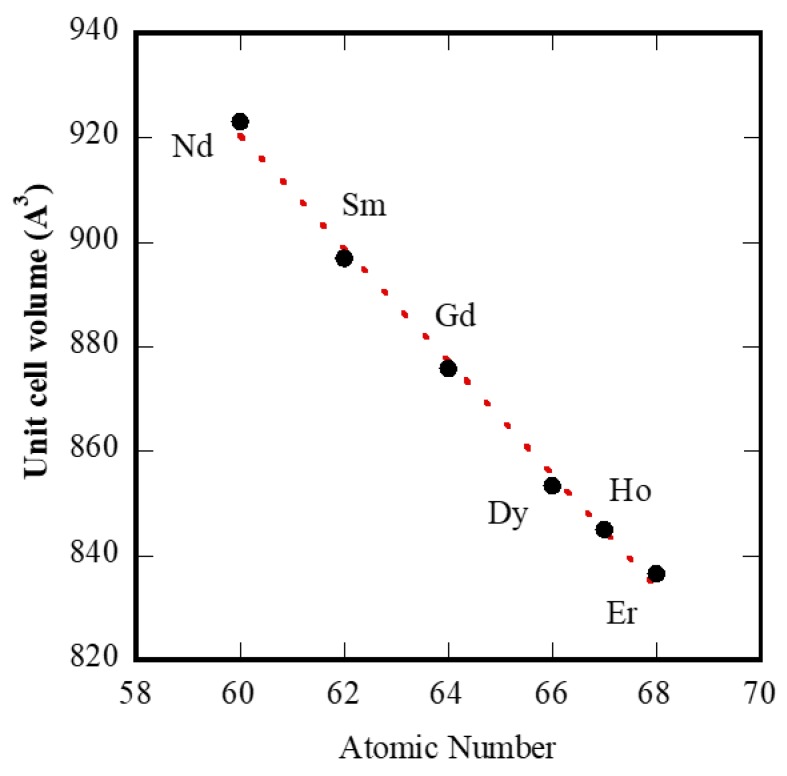
Unit cell volumes of lanthanide carbonates as a function of atomic number.

Therefore, it appears clear that the transformations in the hydrothermal treatments lasting 8 h gradually change, moving along the rare-earth group, even if some “discontinuities” in the behavior emerge, as seen between Nd and Sm, and between Er and Yb; and possibly also between Sm and Gd. In other words, we can identify a first group (from La to Nd) with the same behavior even if with a gradually changing rate of transformation; a second group (from Sm, or Gd, to Er) in which, again, there is the same behavior with a gradually changing rate; and a third group, formed only by Yb in our experimentation, with a further different behavior. The division of lanthanides into several groups based on the formed products and the formation kinetics is well known. For example, Mai et al. [39] reported that in the synthesis of NaREF_4_ three different groups of lanthanides were revealed: (i) Pr and Nd, (ii) from Sm to Tb, and (iii) from Dy to Lu and Y. On the other hand, the double-double effect, based on quantum mechanical interelectronic repulsion energy of the f-electrons [40] from a phenomenological point of view results in the main division of the whole group of f-electron elements (f^0^–f^14^) into two subgroups, f^0^–f^7^ and f^7^–f^14^, and in the further internal division of each of the two subgroups by the f^3^–f^4^ and f^10^–f^11^ pair, respectively [41]. The presence of this effect can be highlighted by a plot of a suitable property vs. atomic number. Properties such as free energies of complex formation and extraction, enthalpies and entropies of extraction, free energy of crystallization, free energy of hydration of lanthanide ions, and unit cell volumes of lanthanide and actinide compounds are all used to show this effect [40,42]. Considering our experimental results, the last property can be used for testing the double-double effect, at least for “tengerite-(RE)” with RE from Nd to Er. Thus, in Figure 9 we have replotted Figure 4, but removing Y, which is not a lanthanide, and using the atomic number as the x-axis. The plot is a nearly perfect line and is very different from the unit cell volumes data reported in [43] related to several rare-earth compounds. Our results seem to suggest that there is no “discontinuity” inside the series of lanthanide carbonates synthesized via hydrothermal treatment, whereas on the contrary, the “discontinuities” appear with reference to the formed phase, as happens in correspondence with Nd and in correspondence with Er. These further “discontinuities” were also confirmed by Müller et al. [44] who claimed that sudden changes in properties and stability constants of lanthanide-coordination compounds were not only found around gadolinium but were also reported to occur with neodymium or erbium.

Considering that the Y-based precursor behaves analogously to rare-earth with similar ionic radius, one can suppose that the size of the cation RE^3+^ plays a crucial role in the hydrothermal synthesis of rare-earth carbonates. In other words, we could explain the different results of the hydrothermal treatments as a function of the rare-earth on the basis of the well-known lanthanide contraction law. In fact, the larger the size of the cation RE^3+^, the faster the kinetics of the hydrothermal transformations, which should result in to the hydroxycarbonates formation. In this regard, Yb (having the smallest size among the studied rare-earths), exhibits the slowest kinetics and, in fact, the product remains significantly amorphous even after aging for 210 h. Furthermore, Er crystallization (having the second smallest cation size) was more difficult than the other rare-earths with type-C behavior, requiring 8 h of treatment for its full crystallization. However, an opposite behavior occurred for La, Ce and Pr, i.e., the largest cations, in which the hydroxycarbonates were formed within 8 h, or even less. In conclusion, the limit value for the ionic radius of RE^3+^ to ensure full crystallization in the 8 h hydrothermal treatment was 0.1 nm.

A possible explanation of this strong effect of the cationic radius on the hydrothermal behavior could lie in the increasing covalent (less basic) character of the bonds that occurs when decreasing the cationic size of the rare-earths, according to the well-known Fajans’ rule. A higher covalent character of bonds, strictly related to both cation size and charge, could hinder the dissolution–precipitation mechanism, which is the basis of the hydrothermal transformations, thus making achievement of the equilibrium condition, i.e., the formation of the hydroxycarbonates, more difficult.

Therefore, in order to explain the behavior during the hydrothermal treatment, not only the behavior of the cation RE^3+^ in solution should be considered, but also the dissolution of the metastable solid phases formed (amorphous and crystalline).

## 5. Conclusions

The hydrothermal aging of amorphous precursors formed by precipitation with ammonium carbonate of rare-earth nitrates leads to different results according to the lanthanide contraction law. In this work, we have found the suitable chemical–physical conditions for the synthesis of a rare-earth carbonate with a tengerite-type structure, with the chemical formula RE_2_(CO_3_)_3_·2-3H_2_O, and characterized by acicular-like morphology, for rare-earths from Nd to Er. By considering that the stable form of rare-earth carbonates in aqueous environment of the hydrothermal treatment is the hydroxycarbonate, we have showed that with the decrease of the rare-earth ionic radius, the kinetics of this transformation slow down. Additionally, albeit the hydrothermal treatment was carried out at 120 °C, for several rare-earths the hydroxycarbonate was not formed at all, even in the case of prolonged treatments. In particular, for rare-earths of larger size (La, Ce, Pr), the hydroxycarbonates were readily formed after a short time. The same final products were also obtained for rare-earths of intermediate size (Nd), even though longer treatments were needed. In contrast, for the smaller size rare-earths (Y is included in this class), the carbonate in the form of tengerite was quickly formed (i.e., within 2 h), with it being stable after very long periods. Therefore, it is possible to synthesize carbonates of various rare-earths in this way, with a simple and cheap process in an aqueous environment. It is probable that these are metastable phases, but that their transformation kinetics are extremely slow because of the chemical properties induced by their smaller ionic radius. Finally, for the smallest rare-earth, i.e., Yb, the kinetics of dissolution are so slow that Yb-based carbonate is not able to fully crystallize in the chemical–physical conditions of the adopted hydrothermal treatment, even after very long durations. Only an incipient crystallization (i.e., not yet fully complete) of the Yb-based carbonate occurred with a far more concentrated solution and longer duration of treatment.

## Figures and Tables

**Table 1:** Lattice parameters and unit cell volume of rare-earth carbonates with tengerite-type structure.

Parameters	Nd8h	Sm8h	Gd8h	Dy8h	Y8h	Ho8h	Er8h
a (nm)	0.62634(1)	0.62129(1)	0.61618(4)	0.61108(5)	0.60893(7)	0.60904(2)	0.60689(9)
b (nm)	0.94542(5)	0.93644(4)	0.92922(6)	0.92109(3)	0.91735(9)	0.91818(6)	0.91519(4)
c (nm)	1.55881(2)	1.54165(2)	1.52944(6)	1.51600(8)	1.51102(2)	1.51100(4)	1.50603(5)
Cell Volume (nm^3^ × 1000)	923.06(9)	896.93(9)	875.72(2)	853.30(9)	844.07(8)	844.97(5)	836.49(8)

**Table 2:** Lattice parameters and unit cell volume of RECO_3_OH.

Parameters	La8h	Ce8h	Pr8h
a (nm)	1.26415(9)	1.25302(4)	1.24543(9)
c (nm)	1.00053(9)	0.99603(7)	0.99183(6)
Cell Volume (nm^3^ × 1000)	1384.74(1)	1354.33(0)	1332.34(2)
